# Clinical Significance of microRNA-196b-5p in Hepatocellular Carcinoma and its Potential Molecular Mechanism

**DOI:** 10.7150/jca.29293

**Published:** 2019-08-29

**Authors:** Lu Zhang, Bin Luo, Yi-wu Dang, Rong-quan He, Zhi-gang Peng, Gang Chen, Zhen-bo Feng

**Affiliations:** 1 Department of Pathology, First Affiliated Hospital of Guangxi Medical University, No. 6 Shuangyong Road, Nanning, Guangxi Zhuang Autonomous Region 530021, P. R. China; 2 Department of Medical Oncology, First Affiliated Hospital of Guangxi Medical University, No. 6 Shuangyong Road, Nanning, Guangxi Zhuang Autonomous Region 530021, P. R. China

**Keywords:** microRNA-196b-5p, hepatocellular carcinoma, quantitative reverse transcription and polymerase chain reaction, bioinformatics

## Abstract

**Objective**: To enquire into the clinical significance and potential molecular mechanism of microRNA (miRNA)-196b-5p in hepatocellular carcinoma (HCC).

**Methods**: Quantitative reverse transcription and polymerase chain reaction (qRT-PCR) were utilized to examine miR-196b-5p expression level in 67 HCC paraffin embedded tissues and corresponding adjacent tissues. Correlations of miR-196b-5p expression level with clinicopathological characteristics were analyzed in our study. The expression level and clinical significance of miR-196b-5p in HCC were also evaluated in The Cancer Genome Atlas (TCGA) and Gene Expression Omnibus (GEO) database. We made predictions of the target genes of miR-196b-5p by twelve online software and then selected genes predicted by at least 5 software. Subsequently, in order to obtain the potential target genes of miR-196b-5p, we overlapped the predicted target genes and down-regulated mRNAs in HCC based on TCGA database. Then, we performed the Gene Ontology (GO) and the Disease Ontology (DO) functional annotation, Kyoto Encyclopedia of Genes and Genomes (KEGG) pathway enrichment analysis and Protein-Protein Interaction (PPI) network construction of those miR-196b-5p potential target genes.

**Results**: Higher expression level of miR-196b-5p was seen in HCC tissues than in the corresponding adjacent tissues based on qRT-PCR (*P* = 0.0007). The expression level of miR-196b-5p was linked with tumor size (*P* = 0.03), tumor node (*P* = 0.024), vascular invasion (*P* = 0.029) and capsular invasion (*P* = 0.026) in HCC patients. Comprehensive meta-analysis of miR-196b-5p expression based on TCGA, GEO and qRT-PCR verified that higher expression level of miR-196b-5p was observed in HCC tissues than in normal control liver tissues (SMD = 0.56, 95%CI: 0.39-0.72, *P*_heterogeneity_ = 0.275, I^2^ = 18.3%). GO annotation revealed that the top terms in biological process, cellular component and molecular function were single-organism catabolic process, neuronal cell body and transmembrane receptor protein kinase activity, respectively. The most relevant disease in DO annotation was arteriosclerosis. The tryptophan metabolism pathway ranked first in KEGG pathway enrichment analysis. The PPI network showed that IGF1, FOXO1, AR and FOS were mostly likely to become the core genes of miR-196b-5p potential target genes, which however required further experiments for validation.

**Conclusion**: The miR-196b-5p was observed to show higher expression in HCC tissues than in normal control liver tissues. Moreover, the miR-196b-5p expression level had correlations with the clinicopathological parameters such as vascular invasion of HCC, but the molecular mechanisms of miR-196b-5p in HCC still need further elucidation and verification.

## Introduction

Hepatocellular carcinoma (HCC) represented approximately 85-90% of the primary hepatic cancer. In China, HCC ranked fourth in the morbidity rate and third in mortality rate among all the malignancies 1, 2. The number of HCC patients increased by over 400 thousand annually, most of whom were diagnosed at the advanced stage, which resulted in the unpleasant prognosis and relatively low 5-year survival rate 3-5. Notably, Guangxi is a region with a high incidence of HCC 6, 7. Currently, the main treatment for HCC was operation, but the operation failed to achieve satisfactory clinical cure rate and long-term survival rate 8, 9. Therefore, the major concern is to seek novel treatments for HCC. Recently, the molecularly targeted therapy has been rising as a new approach to dealing with cancers 10-12. The initiation and progression of HCC was considered a continual and complex process that involved multiple factors and stages of evolution, in which abnormal changes were detected in the structures and expressions of a large number of coding or non-coding-RNAs. In recent years, the research on the non-coding RNAs has refreshed our knowledge on the initiation and development of HCC, the early diagnostic markers and the novel therapeutic targets 13-15.

The microRNA (miRNA), a sort of non-coding RNA (involving around 20-22 nucleotides) without the protein coding function, affects RNA silencing and post-transcriptional regulation of gene expression 16, 17. Several researches indicated that miRNA closely correlated to the onset and development of tumors 18, 19. Studies by deep sequencing and gene microarrays also showed that a number of miRNA was aberrantly expressed in cancer cell lines and tumor tissues, and they participated in the biological process such as the tumor onset, development, metastasis and so on, influencing the growth and proliferation of cancer cells, invasion, metastasis, apoptosis, autophagy, etc. 20-24. Thereby, the identification of miRNA in the initiation and progression of tumors could assist us to investigate the mechanism of tumor initiation and progression, and to seek the novel diagnostic markers and therapeutic targets 25-33.

The miR-196b-5p, which is categorized into the miR-196b family, is located on human chromosome 7p15.2. Previous studies had shown that overexpressed miR-196b-5p was observed in colorectal carcinoma 34 and gastric carcinoma 35. In the colorectal cancer, miR-196b-5p could regulate the invasion as well as the metastasis of colorectal cancer cells by targeting HOXB7 and GALNT5 36. In terms of the research on miR-196b in the HCC, only Shen et al. 37 detected its overexpression in HCC, and no studies was found on the clinical value of miR-196b-5p in HCC and its mechanism. By quantitative reverse transcription and polymerase chain reaction (qRT-PCR), this study examined the expression of miR-196b-5p in the HCC tissues and the adjacent paraffin embedded tissues, and explored its clinical significance as well. Besides, for the purpose of investigating the expression of miR-196b-5p and its clinical significance in HCC, we took advantage of the RNA sequencing (RNA-seq) data in The Cancer Genome Atlas (TCGA, https://cancergenome.nih.gov/cancer) and microarray data in Gene Expression Omnibus (GEO, https://www.ncbi.nlm.nih.gov/geo/). The target genes of miR-196b-5p would be predicted, and also its molecular mechanism in HCC would be explored.

## Materials and Methods

### Tissue samples

The researchers collected 67 cases of HCC tissues and 67 cases of corresponding adjacent paraffin embedded tissues from The First Hospital Affiliated to Guangxi Medical University between Jan. 1, 2015 and May 1, 2016. The present study was approved by the Research Ethics Committee of the First Affiliated Hospital of Guangxi Medical University (Nanning, China), and written informed consent was obtained from all patients.

### RNA extraction and qRT-PCR

According to the instruction of E.Z.N.A.TM FFPE RNA Kit of Omega Bio-Tek, we extracted the total RNA of 67 cases of HCC tissues and their corresponding adjacent tissues. The reverse transcription was conducted with miRNA 1st Strand cDNA Synthesis Kit (by stem-loop) of Vazyme Biotech Co.,Ltd. The relative quantification of miR-196b-5p expression was performed by Applied Biosystems PCR7500, with the reagent being the miRNA Universal SYBR® qPCR Master Mix of Vazyme. The sequencing of miR-196b-5p primers included F:5' -GCGCGTAGGTAGTTTCCTGTT-3', R:5' - AGTGCAGGGTCCGAGGTATT-3. The sequencing of endogenous reference genes primer U6 was F: 5'-CTCGCTTCGGCAGCACA-3', R: 5'- AACGCTTCACGAATTTGCGT-3'. The expression value was calculated with 2^-ΔCt^ method.

### The collection of RNA sequencing data in TCGA

By Xena Public Data Hubs (http://xena.ucsc.edu/public-hubs/), the expression profiling of mature miRNA in HCC was acquired from TCGA, in which the miR-196b-5p was selected, including 369 cases of HCC tissues and 49 cases of normal liver tissues. In addition, the clinicopathologic parameters of HCC were downloaded from TCGA in order to evaluate their relationships with miR-196b-5p 38.

### The collection of microarrays in GEO

The researchers retrieved the miRNA microarrays related to HCC in GEO (https://www.ncbi.nlm.nih.gov/geo/) until Nov. 17, 2017 39. The search strategy was (hepatocellular OR liver OR hepatic) AND (miRNA OR microRNA). The standards for inclusion of the HCC related microarrays were as follows: (1) the cancer samples were diagnosed with HCC; (2) each microarray contained HCC cohort and the control; (3) the expression profiling of miR-196b-5p was available; (4) the species was homo sapiens. Those microarrays were excluded if (1) they failed to provide the expression profiling of miR-196b-5p; (2) they did not involve the controls; (3) their species were animals.

### The prediction of target genes of miR-196b-5p

The twelve online software were linked by miRWalk 2.0 (http://zmf.umm.uni-heidelberg.de/apps/zmf/mirwalk2/), including miRWalk, Microt4, miRanda, mirBridge, miRDB, miRMap, miRNAMap, Pictar2, PITA, RNA22, RNAhybrid and Targetscan, all of which could facilitate the forecast of target genes of miR-196b-5p. Afterwards, genes predicted by at least 5 software were preferred as the predicted genes of miR-196b-5p. We combined those predicted genes and the lowly-expressed differential genes analyzed by R language in TCGA, and selected the overlapping genes as the possible target genes of miR-196b-5p 40.

### Annotation, pathway enrichment analysis and Protein-Protein Interaction (PPI) network construction

The David database (https://david.ncifcrf.gov/) was used for annotation and pathway enrichment analysis of the potential target genes of miR-196b-5p, involving the Gene Ontology (GO) annotation and Kyoto Encyclopedia of Genes and Genomes (KEGG) pathway analysis. The GO annotation consists of biological process, cellular component and molecular function. Furthermore, the annotation included the Disease Ontology (DO) annotation, of which results could be generated by R 3.4.1. In order for PPI network construction, we researchers uploaded the potential target genes of miR-196b-5p to Search Tool for the Retrieval of Interacting Genes (STRING) (https://string-db.org/cgi/input.pl), with the confidence score > 0.7 39, 41, 42.

### Statistical analysis

In this study, we researchers examined the expression of miR-196b-5p with qRT-PCR, and calculated the expression value using 2^-△CT^ method: △CT = CT_miR-196b-5p_-CT_U6_. Each sample in triplicate was used in qRT-PCR, and the average CT value was determined. The expressed miR-196b-5p was distributed in skewness in the cancer and adjacent tissues. The Wilcoxon was applied for the test, and the median value was used as a cut-off for the high and low expressions of miR-196b-5p in HCC tissues. The categorical data, like the relationships of expressed miR-196b-5p with the clinicopathologic parameters, were dealt with the Fisher's exact test or χ^2^, whereas the ranked data were processed with the Kruskal-Wallis H test.

In TCGA and GEO, the expressed miR-196b-5p was shown in normal distribution in tumor and adjacent tissues, and we used the Student's T test to estimate the mean of the expression in the two cohorts. GraphPad Prism Version 5.0 (GraphPad Software, San Diego CA, USA; https://www.graphpad.com) was applied to draw the scatter diagram. In addition, the researchers utilized the receiver operating characteristic curve (ROC curve) to assess the capability of expressed miR-196b-5p to distinguish the cancer from non-cancer tissues, and the area under the curve (AUC) could be applied to quantitatively measure the capability, with great value indicating the great capability. We researchers took advantage of SSPS 22.0 to draw the individual ROC curve. In addition, Stata Version 12.0 was used to draw the summary ROC (SROC) curve which could systematically assess the capability of miR-196b-5p expression to distinguish the cancer from non-cancer tissues.

The Standard mean difference (SMD) and 95% confidence interval (95% CI) were calculated to scrutinize the expression of miR-196b-5p in HCC and the normal tissues. The heterogeneity of the Meta-analysis was represented with chi-square test of Q or inconsistency index (I^2^). When the *P* value < 0.05 or I^2^ > 50%, the heterogeneity obviously existed in the Meta-analysis, and then the random effects model would be used. If the *P* > 0.05 or I^2^ < 50%, we found no remarkable heterogeneity in the Meta- analysis, thereby employing the fixed effects model. We applied the Stata Version 12.0 (StataCorp, College Station, TX, USA; http://www.stata.com) to draw the forest plots and conducted sensitivity analysis of the Meta results. Following the sensitivity analysis, we excluded the studies beyond the base line, and then drew the forest plots again. On the condition that the SMD or 95%CI scarcely varied and no obvious heterogeneity was found, the results of Meta-analysis seemed valid and reliable. Besides, the publication bias of the Meta-analysis was evaluated through Egger's and Begg's tests, and then drew the funnel plots. The* P* value > 0.05 (of Egger's and Begg's tests) would mean no publication bias existed.

## Results

### The expression of miR-196b-5p in HCC tissues and in corresponding adjacent tissues

**Figure 1:**
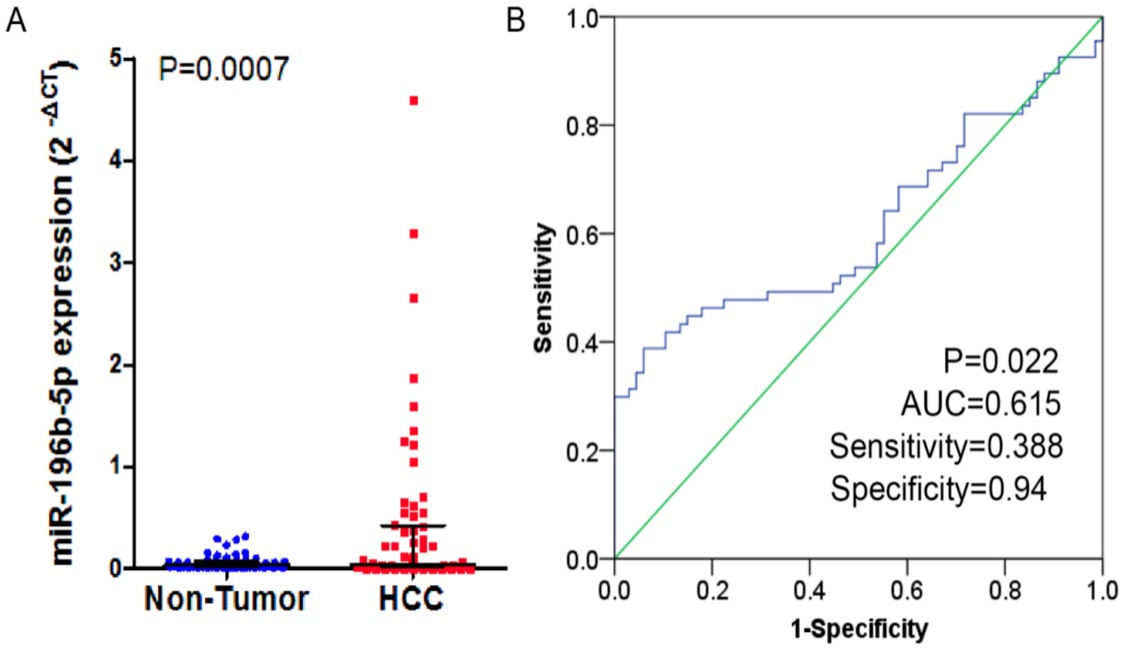
The expression level and ROC curve analysis of miR-196b-5p in HCC based on qRT-PCR. **A**: miR-196b-5p expression level in HCC was higher than in adjacent tissues. **B**: ROC curve analysis of miR-196b-5p for discriminating HCC from adjacent tissues.

In the study, 41 of 67 cases (61.2%) showed that higher expression of miR-196b-5p was seen in HCC tissues than in the adjacent ones. In these 67 cases, the expressed miR-196b-5p displayed skewed distribution in HCC and the adjacent tissues. In HCC tissues, the median of the relative expression of miR-196b-5p was 0.042, and the inter-quartile range was 0.020-0.404, while in the adjacent tissues, the median value was 0.037 and the inter-quartile range was 0.017-0.072. By Wilcoxon test, we ascertained that the higher relative expression of miR-196b-5p was examined in HCC rather than in adjacent tissues, with statistical significance (*P* = 0.0007) (**Figure 1A**). The ROC curve was applied to assess the ability of miR-196b-5p expression to differentiate between the cancer tissues and the non-cancer ones, of which result revealed that AUC was 0.615 (95%CI: 0.517-0.713, *P* = 0.022), with sensitivity being 0.388 and specificity being 0.94 (**Figure 1B**). The analysis of the relationships between miR-196b-5p expression and the clinicopathologic parameters revealed that the expression was associated with tumor size, nodule number, vascular and capsular invasions (*P* < 0.05) (**Table 1**).

### The clinical significance of miR-196b-5p in HCC in TCGA

**Figure 2:**
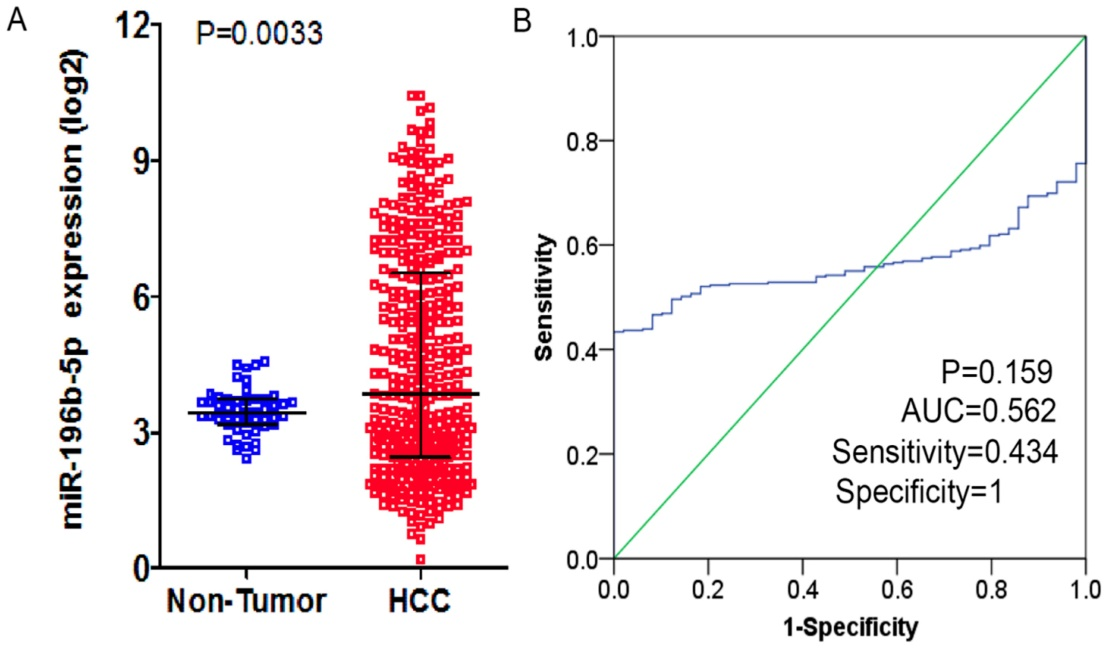
The expression level and ROC curve analysis of miR-196b-5p in HCC based on TCGA database. **A**: miR-196b-5p expression level in HCC was higher than in normal liver tissues. **B**: ROC curve analysis of miR-196b-5p for discriminating HCC from normal liver tissues.

From TCGA database, the researchers downloaded the expression profiling of miR-196b-5p and subsequently compared the 369 cases of HCC tissues and 49 cases of normal liver tissues, finding expression of miR-196b-5p was seen higher in HCC tissues (4.49 ± 2.42) rather than in normal tissues (3.47 ± 0.50), with statistical significance (*P* = 0.0033), (**Figure 2A**). The AUC of miR-196b-5p expression to discriminate cancer tissues from the non-cancer ones was 0.562 (95%CI: 0.511-0.613, *P* = 0.159), with sensitivity and specificity being 0.434 and 1 respectively (**Figure 2B**). The analysis of the relationships between miR-196b-5p expression and the clinicopathologic parameters proved that the expression was in a close association with the sex of patients with HCC, pathologic grading and vascular invasions (**Table 2**).

### The expression of miR-196b-5p in HCC in GEO

**Figure 3:**
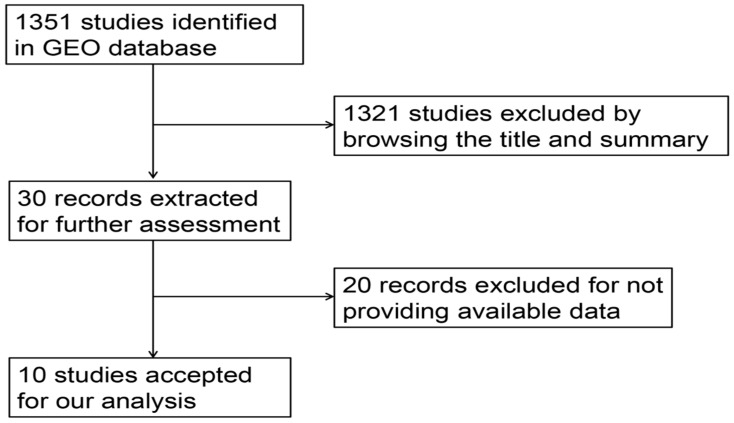
Flow chart of study selection for miR-196b-5p microarray data based on GEO datasets.

**Figure 4:**
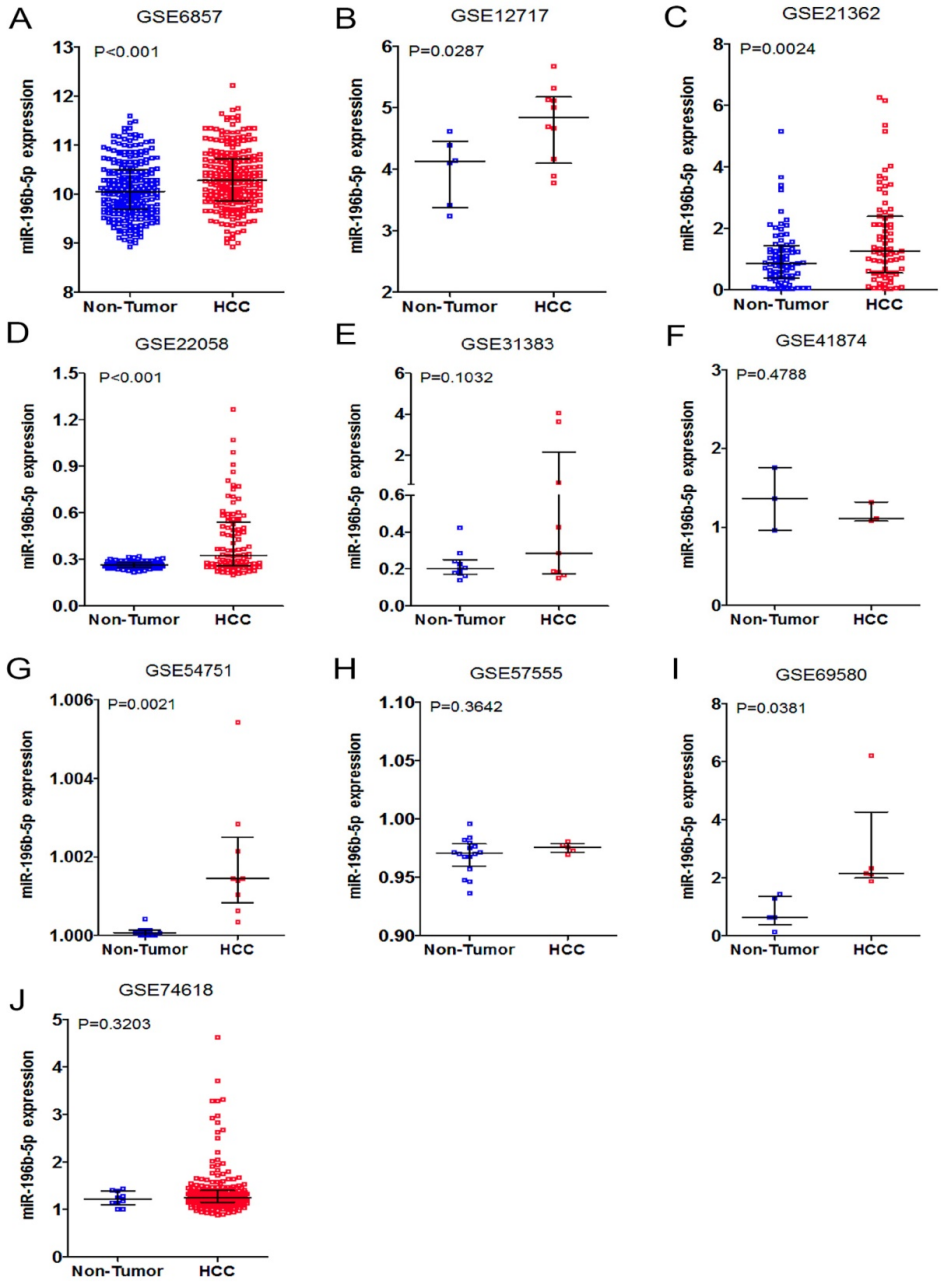
The expression data of miR-196b-5p in HCC in ten microarrays from GEO datasets. **A**: the expression level of miR-196b-5p from GSE6857. **B**: the expression level of miR-196b-5p from GSE12717. **C**: the expression level of miR-196b-5p from GSE21362. **D**: the expression level of miR-196b-5p from GSE22058. **E**: the expression level of miR-196b-5p from GSE31383. **F**: the expression level of miR-196b-5p from GSE41874. **G**: the expression level of miR-196b-5p from GSE54751. **H**: the expression level of miR-196b-5p from GSE57555. **I**: the expression level of miR-196b-5p from GSE69580. **J**: the expression level of miR-196b-5p from GSE74618.

**Figure 5:**
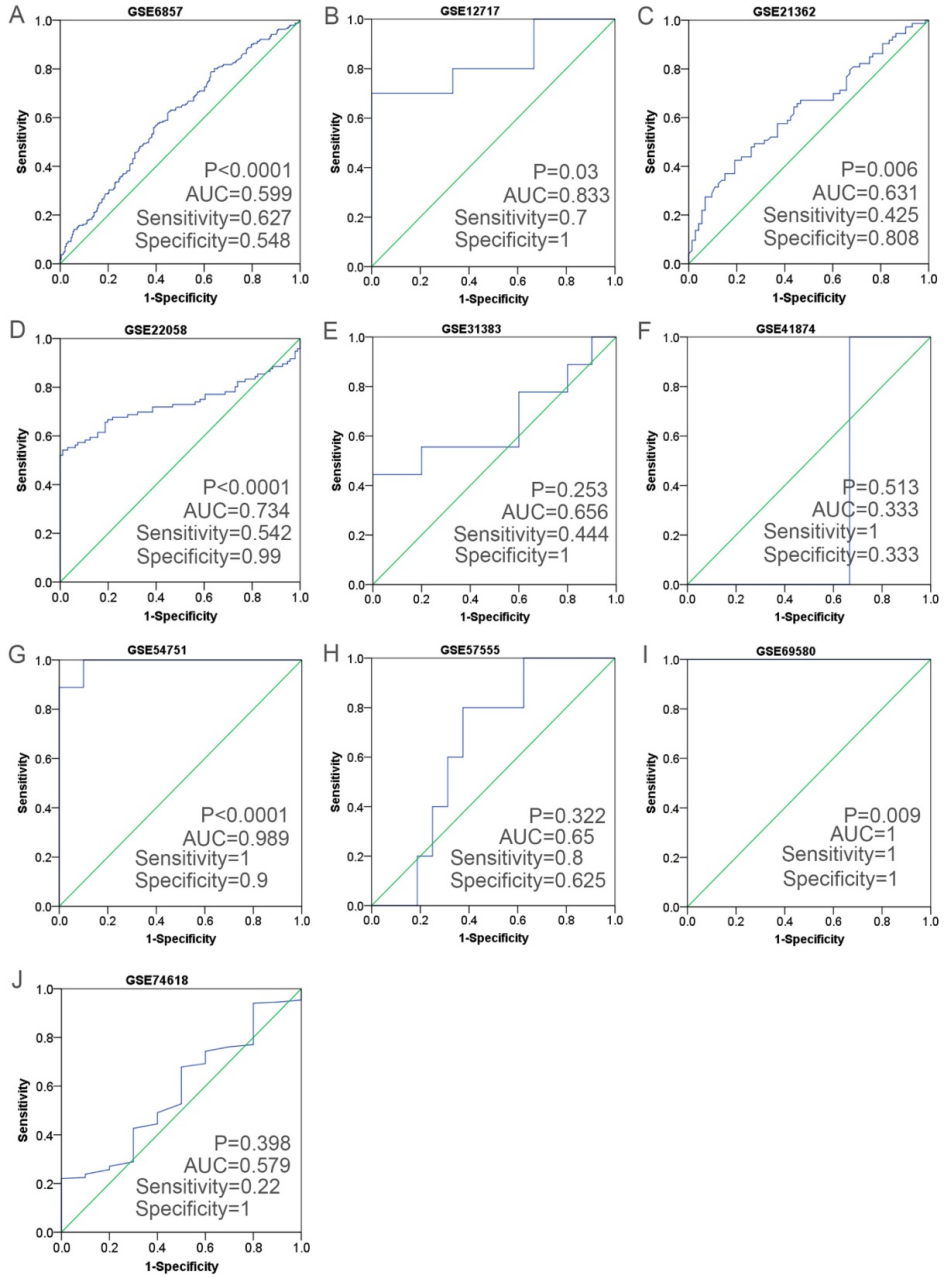
The ROC curves of miR-196b-5p in HCC in ten microarrays from GEO datasets. **A**: the ROC curve of miR-196b-5p from GSE6857. **B**: the ROC curve of miR-196b-5p from GSE12717. **C**: the ROC curve of miR-196b-5p from GSE21362. **D**: the ROC curve of miR-196b-5p from GSE22058. **E**: the ROC curve of miR-196b-5p from GSE31383. **F**: the ROC curve of miR-196b-5p from GSE41874. **G**: the ROC curve of miR-196b-5p from GSE54751. **H**: the ROC curve of miR-196b-5p from GSE57555. **I**: the ROC curve of miR-196b-5p from GSE69580. **J**: the ROC curve of miR-196b-5p from GSE74618.

Having retrieved the GEO database, we included in our research 10 microarrays of miR-196b-5p expression profiling, involving GSE6857, GSE12717, GSE21362, GSE22058, GSE31383, GSE41874, GSE54751, GSE57555, GSE69580 and GSE74618. The retrieval process was displayed in **Figure 3**. The basic features of these 10 microarrays were shown in **Table 3**. In 6 microarrays, compared with the normal tissues, miR-196b-5p was expressed higher in HCC tissues (GSE6857, GSE12717, GSE21362, GSE22058, GSE54751 and GSE69580) (**Figure 4A-D, G, H**). However, no statistical significance was found in miR-196b-5p expression in HCC and normal liver tissues (GSE31383, GSE41874, GSE57555, GSE74618) (**Figure 4 E, F, H, J**). The ROC curves of each microarray were displayed in **Figure 5**.

### The analysis of miR-196b-5p expression in HCC by combination of TCGA, GEO and qRT-PCR results

**Figure 6:**
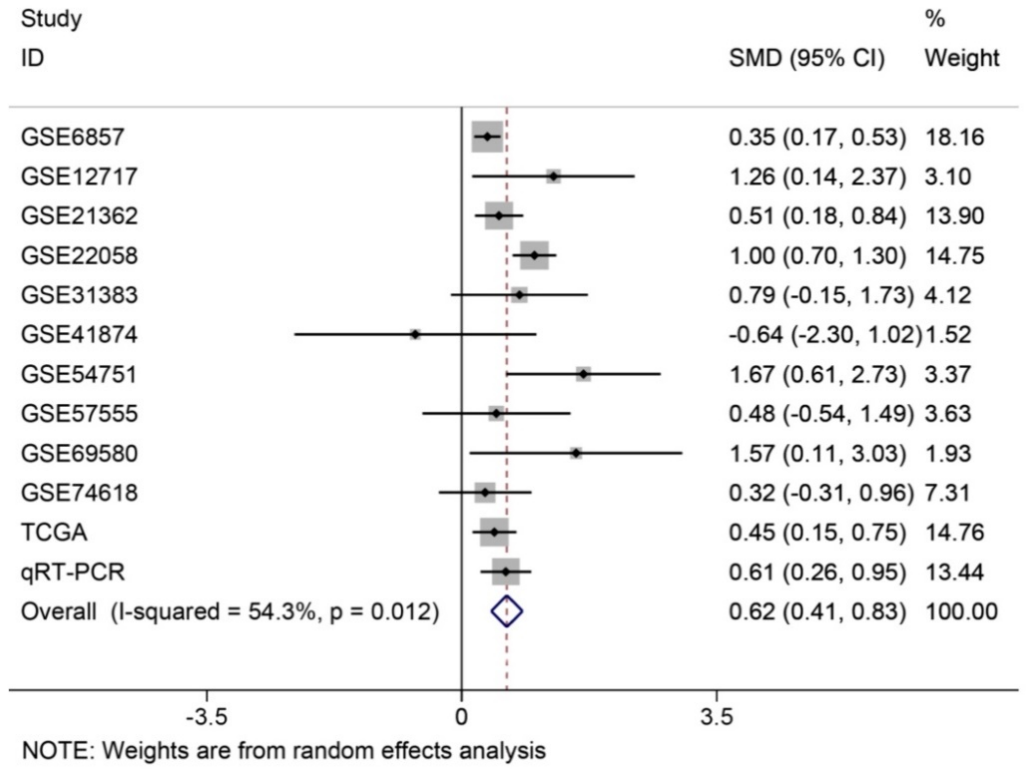
Forest plot of studies evaluating standard mean difference of miR-196b-5p expression between HCC group and non-tumor group based on TCGA, GEO and qRT-PCR.

**Figure 7:**
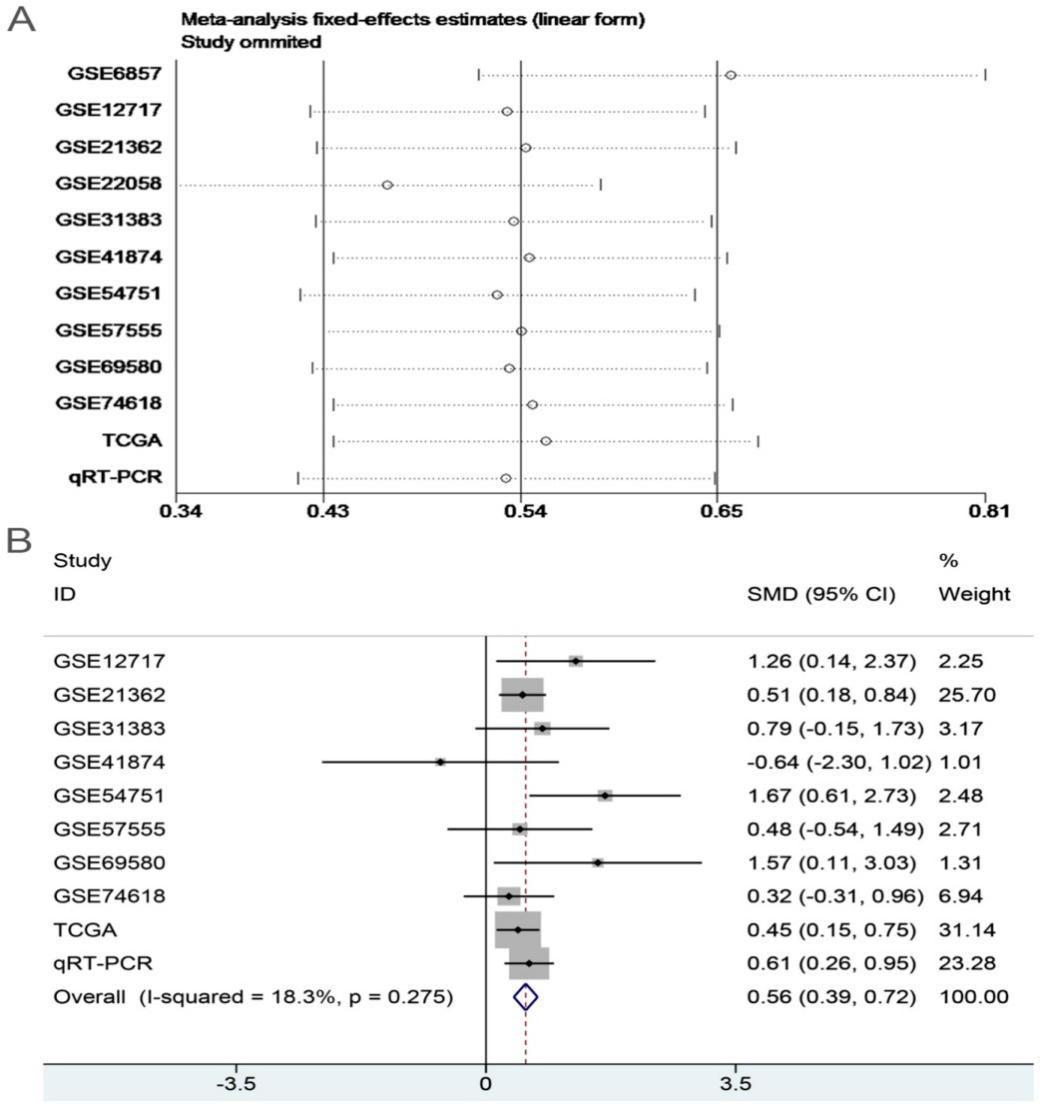
Sensitivity analysis and the forest plot after sensitivity analysis. **A**: sensitivity analysis of Meta-analysis of the expression level of miR-196b-5p in HCC group and in non-tumor group based on TCGA, GEO and qRT-PCR. **B**: forest plot of miR-196b-5p expression between HCC group and non-tumor group after removing the study of GSE6857 and GSE22058.

**Figure 8:**
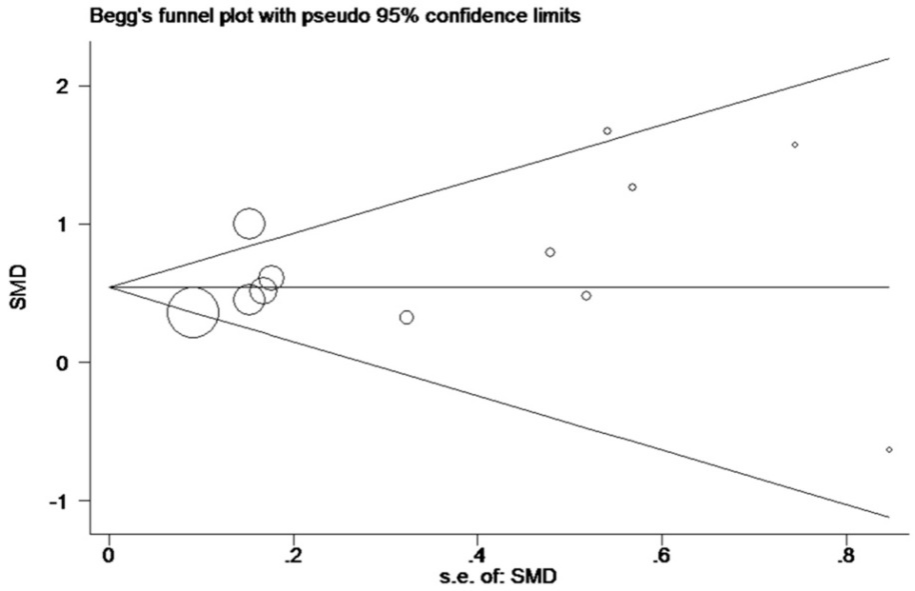
Funnel plot for publication bias test after Meta-analysis of the expression level of miR-196b-5p based on TCGA, GEO and qRT-PCR.

In order to systematically and holistically analyze the miR-196b-5p expression, we researchers combined the results of TCGA, GEO and qRT-PCR for Meta-analysis, which contained 1105 cases of HCC tissues and 586 cases of normal liver tissues. The Meta-analysis showed SMD = 0.62 (95%CI: 0.41-0.83); since the SMD > 0 and 95%CI did not cover 0, it was demonstrated that miR-196b-5p displayed was expressed higher in HCC tissues rather than in normal liver tissues (**Figure 6**). The heterogeneity test revealed that remarkable heterogeneity was observed in Meta-analysis of miR-196b-5p expression (*P*_heterogeneity_ = 0.012, I^2^ = 54.3%; **Figure 6**), so random effects model was preferred. In the sensitivity test, after excluding GSE6857 and GSE22058, we discovered SMD = 0.56 (95%CI: 0.39-0.72), and failed to detect obvious heterogeneity (*P*_heterogeneity_ = 0.275, I^2^ = 18.3%;** Figure 7A, B**). Egger's test showed *P* = 0.230, and Begg's test showed *P* = 0.304; in addition, the funnel plots were distributed in symmetry, suggesting that we failed to detect remarkable publication bias in the Meta-analysis of miR-196b-5p expression (**Figure 8**).

**Figure 9:**
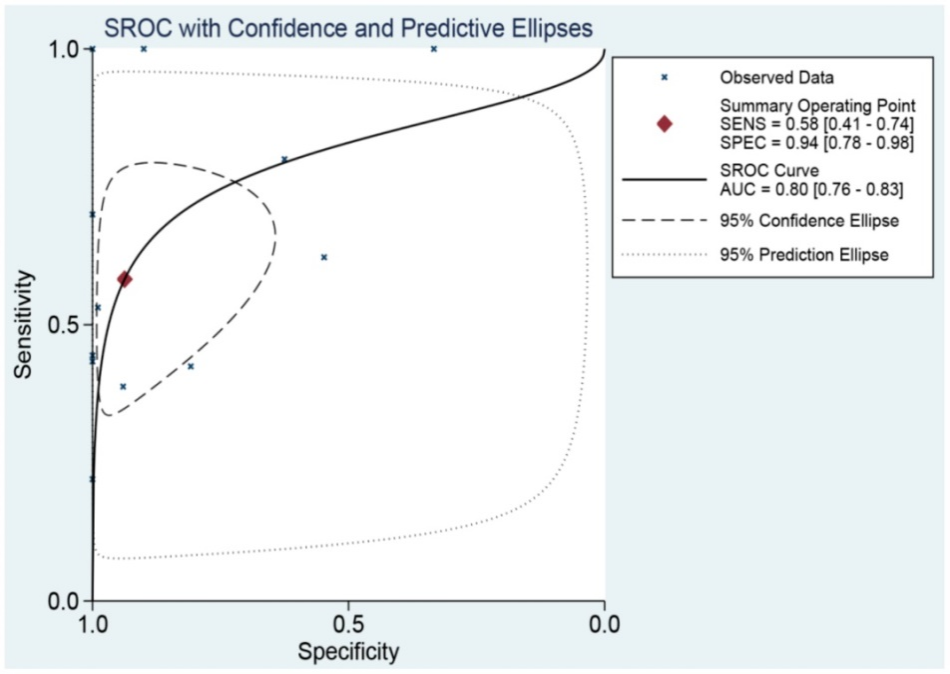
SROC curve analysis of miR-196b-5p for discriminating HCC from normal liver tissues based on TCGA, GEO and qRT-PCR.

The SROC curve was applied to analyze and calculate the AUC and 95%CI in order to systematically evaluate the ability of miR-196b-5p expression to distinguish the cancer tissues from the non-cancer ones. As **Figure 9** illustrated, the total AUC of miR-196b-5p expression was 0.80 (95%CI: 0.76-0.83), with the sensitivity being 0.58 (95%CI: 0.41-0.74) and specificity being 0.94 (95%CI: 0.78-0.98).

### The potential target genes of miR-196b-5p

**Figure 10:**
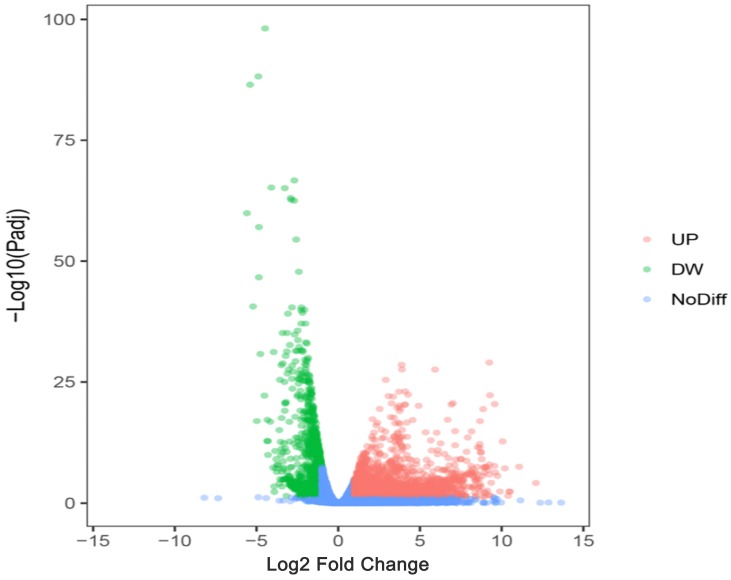
The volcano plot of mRNA expression in HCC based on TCGA database. The pink dots represent up-regulated genes, the green dots represent down-regulated genes and the blue dots are non-differentially expressed genes.

We researchers predicted the target genes of miR-196b-5p with the help of twelve online softwares (miRWalk, miRanda, Microt4, mirBridge, RNA22, miRMap, miRDB, miRNAMap, Pictar2, PITA, RNAhybrid and Targetscan), and then selected 1955 genes that were predicted by at least 5 software. Subsequently, we combined these 1955 genes and 1123 down-regulated mRNAs in TCGA (Log2 Fold Change < -1, P < 0.05) (**Figure 10**), acquiring 107 overlapping potential target genes (**Table 4**).

### Annotation, pathway enrichment analysis and PPI network construction

**Figure 11:**
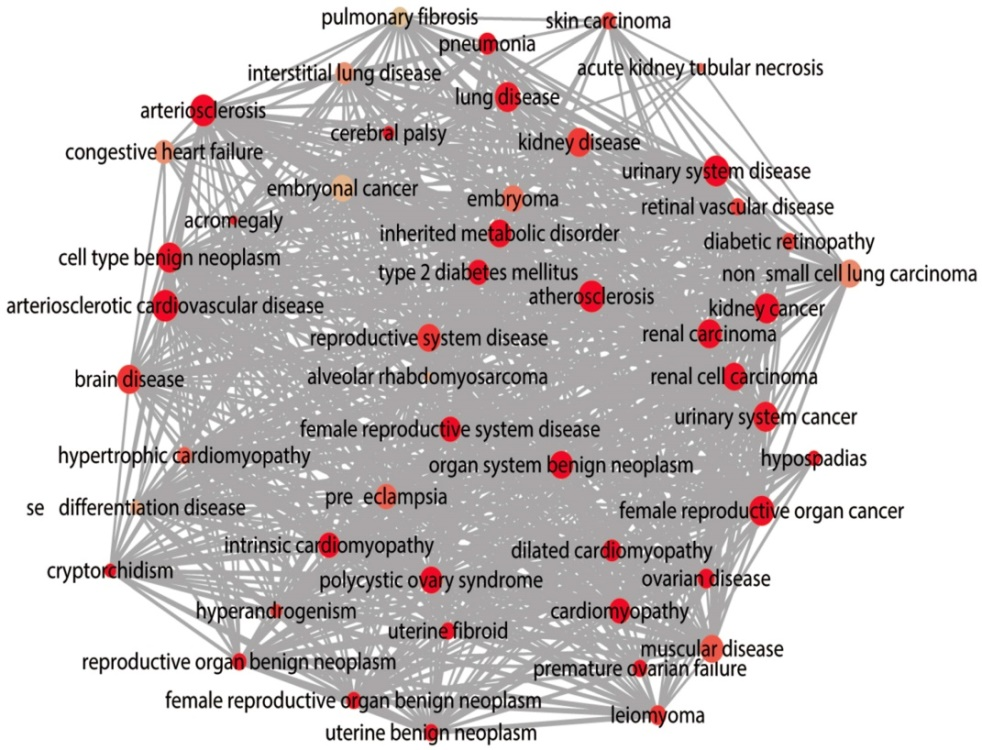
DO functional annotation of the 107 potential target genes of miR-196b-5p, circles represent diseases and edges represent disease-disease associations.

**Figure 12:**
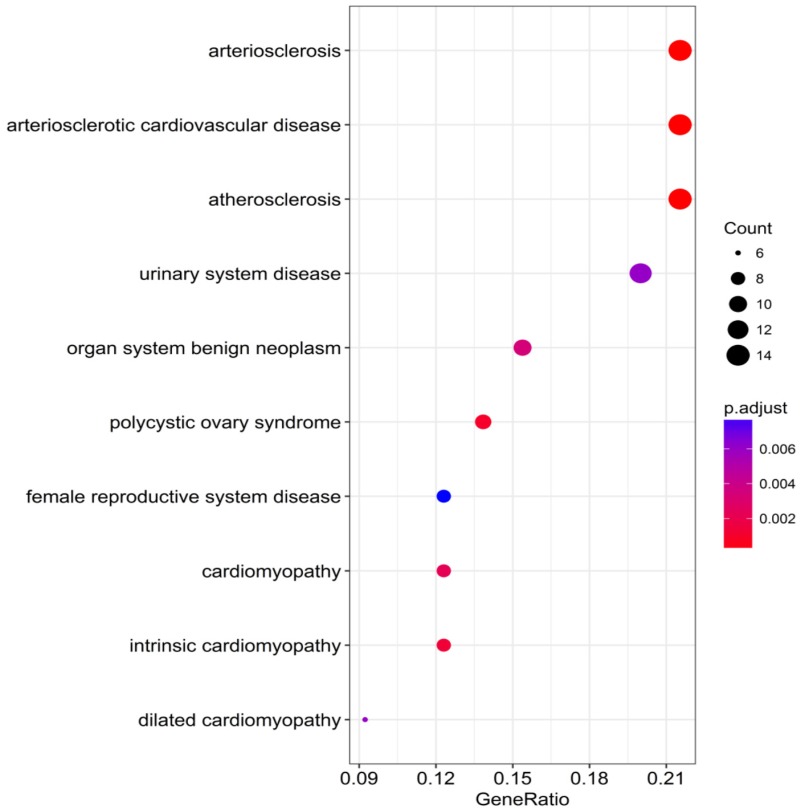
Top 10 DO functional annotation terms of the 107 potential target genes of miR-196b-5p, color tints represent the P-values and size of the circle represent the number of selected genes in the pathway.

**Figure 13:**
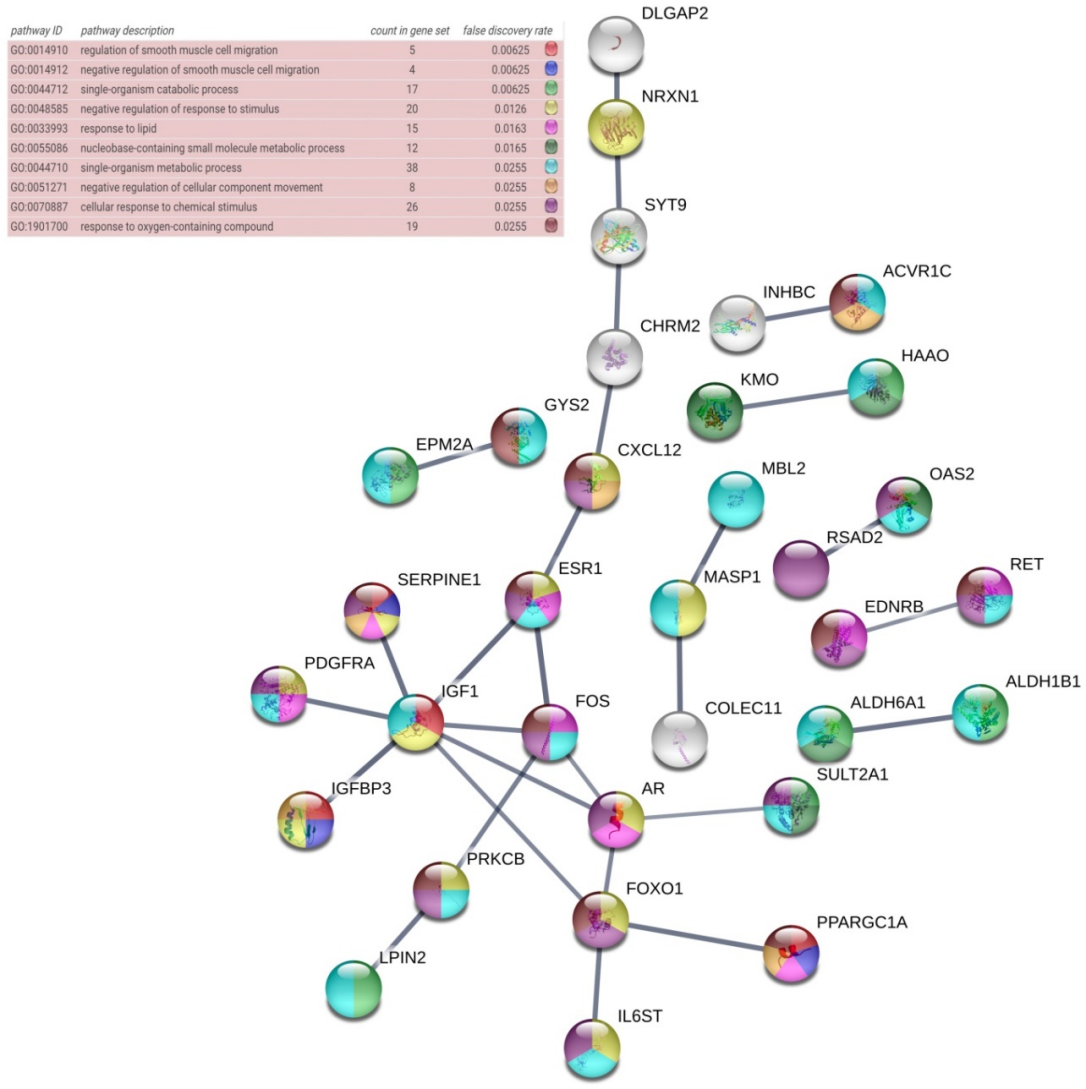
PPI network of the 107 potential target genes of miR-196b-5p constructed by STRING online database, nodes represent proteins and edges represent protein-protein associations.

The DAVID database was utilized for GO annotation and KEGG pathway enrichment analysis for the purpose of exploring the functions of these 107 possible target genes of miR-196b-5p as well as the relevant molecular mechanism (**Table 5**). The GO analysis displayed that in biological process the potential target genes mainly participated in single-organism catabolic process, response to external stimulus, single-organism metabolic process, etc. In cellular component, these potential target genes were mostly enriched in neuronal cell body, cell periphery and extracellular space. In molecular function, the potential target genes largely took part in the molecular process like transmembrane receptor protein kinase activity, 3',5'-cyclic-AMP phosphodiesterase activity, growth factor binding and so on. The KEGG analysis uncovered that the potential target genes were chiefly involved in pathways of tryptophan metabolism, pathways in cancer, complement and coagulation cascades, beta-alanine metabolism, prostate cancer, morphine addiction and so on. Moreover, DO annotation of the potential target genes were conducted via R 3.4.1 (**Figure 11**), and the top ten terms of DO annotation (based on the P value) were listed in **Figure 12**. Also, we constructed the PPI network of these 107 possible target genes of miR-196b-5p by STRING (**Figure 13**).

## Discussion

The miR-196b-5p, which is categorized into the miR-196b family (containing miR-196a-1, miR-196a-2 and miR-196b), is situated on human chromosome 7p15.2, which is situated in the area of homobox (HOX) gene cluster 43, 44. The nucleotide sequence of mature miR-196a-1 is the same as that of mature miR-196a-2, but mature miR-196b and mature miR-196a varied in one nucleotide 44. Abnormally expressed miR-196b was frequently detected in various tumors like head and neck squamous-cell carcinoma (HNSCC), colorectal carcinoma, lung carcinoma, pancreatic cancer, gastric cancer, etc. In HNSCC, the results of Álvarez-Teijeiro et al. suggested that miR-196b exhibited higher expression in HNSCC fresh tissues and paraffin-embedded tissues than in the adjacent ones; in addition, overexpression of miR-196b was detected in the saliva samples 45. In the case of lung cancer, Bai et al. used qRT-PCR to confirm that remarkably lower expression of miR-196b appeared in various lung cancer cell lines (A549, H-1650 and H-1299) than in healthy lung tissue cells (WI-38 and HEL-1) 46. In pancreatic cancer, Wang et al. examined the differentially expressed miRNAs of 20 cases of pancreatic cancer tissues and the non-cancer ones, and acquired 39 lowly expressed and 40 highly expressed miRNAs in the cancer tissues. Subsequently, qRT-PCR was used to verify the top five miRNAs: miR-200c, miR-196b, miR-1, miR-200a and let-7b (based on the P value), and it was uncovered that miR-196b, miR-200a and miR-200c were detected to be highly expressed, miR-1 was seen lowly expressed, whereas the expression of let-7b showed no statistical significance in cancer tissues and the non-cancer ones 47. By ArrayExpress and TCGA, Ren et al. concluded that miR-196b-5p displayed higher expression in colorectal cancer tissues compared with normal colonic mucosa tissues 48. In gastric carcinoma, Lee et al. took advantage of miRNA microarrays to examine 34 cases of gastric cancer tissues and the adjacent ones, afterwards acquiring 5 highly expressed miRNAs in cancer tissues (miR-196b-5p, miR-215, miR-375, miR-1and miR-370) and 5 lowly expressed miRNAs (miR-2861, miR-483-5p, miR-486-5p, miR-622 and miR-149-3p), and finally confirmed that the expressions of miR-196b-5p and miR-375 were consistent with the microarrays 35. Studies above all suggested that the aberrantly expressed miR-196b was closely linked with morbid state.

In this research, we researchers applied qRT-PCR to examine the miR-196b-5p expression in HCC, discovering that miR-196b-5p displayed higher expression in HCC tissues than in the adjacent ones with the assistance of endogenous reference genes primer U6. The analysis of relationships between miR-196b-5p expression and the clinicopathologic parameters revealed that the expression was associated with the tumor size, nodule number, vascular and capsular invasions. Following that, we selected the miR-196b-5p from the expression profiling of mature miRNA in TCGA database, and uncovered that higher expression of miR-196b-5p was observed in HCC tissues than in healthy liver tissues; the expression was connected with the sex of patients, pathologic grading and vascular invasion. Afterwards, we researchers analyzed the miRNA microarrays in GEO, and carried out Meta-analysis of miR-196b-5p by combining the results of qRT-PCR and TCGA sequencing data, which verified that miR-196b-5p was differentially expressed and overexpressed in HCC tissues.

Results above indicated that miR-196b-5p was abnormally expressed in HCC; more importantly, it played a pivotal part in the initiation and progression of HCC. Nonetheless, no studies were found on its influences in HCC and the molecular mechanism. The miRNAs perform their functions by entirely or partly binding to the target genes, thereby resulting in the degradation or translational control of the target genes. Furthermore, miRNA would participate in the biological behaviors such as the proliferation of tumor cells, invasion, metastasis and so on 49-51. The binding of miRNA to target genes had been sufficiently studied, and the prediction of miR-196b-5p target genes was available in many online databases 52. In this study, taking advantage of 12 online software, we researchers predicted the miR-196b-5p target genes, then selecting those genes appearing in at least 5 software. Next, we combined the genes and the down-regulated mRNAs in TCGA, acquiring the 107 overlapping genes that could be used as possible target genes of miR-196b-5p. Previous studies reported that miR-196b-5p could influence the invasion of tumor cells, metastasis, resistance to drugs, etc. by targeting the downstream target mRNAs and relevant signal transduction pathways. For instance, results of Ren et al. suggested that miR-196b-5p could cause the resistance of colon cancer cells to 5-fluorouracil via STAT3 signal transduction pathway 34. Stiegelbauer et al. put forth that invasion of colon cancer cells and distant metastasis could be controlled by miR-196b-5p targeting HOXB7 and GALNT5 36. In order to clarify the molecular mechanism of miR-196b-5p target genes in HCC, we carried out GO and DO annotations, KEGG pathway enrichment analyses, PPI network construction of the 107 potential target genes, which could elucidate the related pathways and possible biological roles of the potential target genes. The GO annotation unveiled that the top-ranked terms in biological process, cellular component and molecular function were single-organism catabolic process, neuronal cell body and transmembrane receptor protein kinase activity, respectively. The most relevant disease in DO annotation was arteriosclerosis. The tryptophan metabolism pathway ranked first in KEGG pathway enrichment analysis. The PPI network showed that IGF1, FOXO1, AR and FOS were mostly likely to become the core genes of miR-196b-5p potential target genes, which required further experiments for validation.

In this study, we, by means of qRT-PCR, TCGA sequencing data and GEO microarrays, confirmed that miR-196b-5p was expressed remarkably higher in HCC tissues rather than in healthy liver tissues. Moreover, miR-196b-5p expression level had correlations with the clinicopathological parameters such as vascular invasion of HCC. GO annotation revealed that the top-ranked terms in biological process, cellular component and molecular function were single-organism catabolic process, neuronal cell body and transmembrane receptor protein kinase activity, respectively. The most relevant disease in DO annotation was arteriosclerosis. The tryptophan metabolism pathway ranked first in KEGG pathway enrichment analysis. PPI network showed that IGF1, FOXO1, AR and FOS were mostly likely to become the core genes of miR-196b-5p potential target genes, which however required further experiments for validation.

## Figures and Tables

**Table 1:** The relationships between miR-196b-5p expression and the clinicopathologic parameters by qRT-PCR

Clinicopathologic parameters		miR-196b-5p expression		P value
		Cases of high expression	Cases of low expression	
Sex	Male	29	27	
	Female	5	6	0.701
Age	<60	27	27	
	≥60	7	6	0.803
Tumor size (cm)	≥5	27	18	
	<5	7	15	0.03*
Tumor nodule	Single	25	31	
	Multiple	9	2	0.024*
Vascular invasion	Yes	17	8	
	No	17	25	0.029*
Capsular invasion	Yes	12	4	
	No	22	29	0.026*
HBV infection	Yes	28	28	
	No	6	5	0.783
HCV infection	Yes	1	1	
	No	31	32	1
Cirrhosis	Yes	19	17	
	No	15	16	0.72
Portal vein tumor thrombus (PVTT)	Yes	5	2	
	No	29	31	0.449
AFP (ng/ml)	≥400	16	11	
	<400	18	21	0.295
nm23	Positive	31	33	
	Negative	3	0	0.248
P53	Positive	25	27	
	Negative	9	6	0.416
P21	Positive	5	3	
	Negative	29	30	0.74
VEGF	Positive	16	17	
	Negative	17	16	0.806
Ki-67	High	18	19	
	Low	14	13	0.8
CD34	High	15	19	
	Low	9	3	0.066
Pathologic grading	I-II	18	21	
	III-IV	16	12	0.375
Child-Pugh class	A	21	18	
	B	3	2	1
BCLC stage	0	0	2	
	A	12	9	
	B	11	7	
	C	1	2	0.356

**Note:** HCC, hepatocellular carcinoma; AFP, α-fetoprotein; nm23, Non-metastasis 23; VEGF, vascular endothelial growth factor; HBV, hepatitis B virus; HCV, hepatitis C virus; BCLC, Barcelona Clinic Liver Cancer; *P<0.05.

**Table 2:** The relationships between miR-196b-5p expression and the clinicopathologic parameters in TCGA

Clinicopathologic parameters		Number of cases	miR-196b-5p expression	P value
			(mean ± SD)	
Tissues	HCC	369	4.49±2.42	0.003*
	Normal controls	49	3.47±0.50	
Sex	Male	250	4.31±2.42	0.035*
	Female	119	4.88±2.40	
Age	<60	170	4.30±2.41	0.168
	≥60	198	4.65±2.43	
Pathologic grading	I~II	228	4.28±2.37	0.041*
	III~IV	137	4.81±2.44	
Stage	I~II	256	4.52±2.44	0.97
	III~IV	87	4.51±2.41	
T	TX	1	4.16	
	T1	180	4.34±2.37	0.524
	T2-4	186	4.62±2.45	
N	NX	111	4.10±2.30	
	N0	253	4.67±2.46	0.078
	N1	4	3.42±1.70	
M	MX	98	4.09±2.29	
	M0	267	4.64±2.47	0.148
	M1	4	4.84±1.09	
Vascular invasion	Yes	110	4.93±2.56	0.023*
	No	204	4.27±2.34	
Cirrhosis	Yes	6	3.44±2.56	0.272
	No	344	4.53±2.39	
Drinking	Yes	117	4.49±2.32	0.908
	No	233	4.52±2.44	
Smoking	Yes	17	4.82±2.57	0.579
	No	333	4.49±2.39	
HBV infection	Yes	107	4.55±2.58	0.828
	No	243	4.49±2.31	
HCV infection	Yes	55	4.53±2.53	0.948
	No	295	4.50±2.37	

**Note:** HCC, hepatocellular carcinoma; TCGA, The Cancer Genome Atlas; HBV, hepatitis B virus; HCV, hepatitis C virus; *P<0.05.

**Table 3:** The basic features of 10 microarrays of expression profiling of miR-196b-5p form GEO database

Series	Platform	Country/Region	Citation	Number of samples		miR-196b-5p expression (Mean ± SD)	
				HCC	Normal control	HCC	Normal control
GSE6857	GPL4700	USA	Budhu et al. (2008)	241	241	10.322±0.607	10.113±0.575
GSE12717	GPL7274	USA	Su et al. (2009)	10	6	4.742±0.629	3.987±0.544
GSE21362	GPL10312	Japan	Sato et al. (2011)	73	73	1.686±1.463	1.053±0.957
GSE22058	GPL10457, GPL6793, GPL9733	USA	Burchard et al. (2010)	96	96	0.416±0.214	0.265±0.020
GSE31383	GPL10122	USA	Wang et al. (2012)	9	10	1.084±1.584	0.223±0.081
GSE41874	GPL7722	Japan	None	3	3	1.171±0.129	1.359±0.397
GSE54751	GPL18262	USA	Shen et al. (2015)	9	10	1.002±0.002	1.000±0.000
GSE57555	GPL18044, GPL16699	Japan	Murakami et al. (2015)	5	16	0.975±0.004	0.969±0.015
GSE69580	GPL10850	Taiwan	None	5	5	2.938±1.832	0.824±0.527
GSE74618	GPL14613	Spain	Villanueva et al. (2016)	218	10	1.380±0.483	1.227±0.158

**Note:** HCC, hepatocellular carcinoma; USA, United States of America; SMD, standard mean difference.

**Table 4:** A total of 107 potential target genes of miR-196b-5p

Gene names						
UROC1	SPATA18	PTPRD	NFAM1	IGFBP3	DMD	BCO2
TRIB1	SOWAHC	PRKCB	NAALADL2	IGF1	DLGAP2	BACH2
TMEM56	SOCS2	PPARGC1A	MTTP	HAAO	CXCL12	AXL
TMEM25	SLC46A3	PLIN2	MMAA	GYS2	CTBS	ATP13A4
TMEM220	SLC41A2	PLCXD3	MEGF10	GJB2	CR1	ATP11C
TGFBR3	SLC38A4	PDGFRA	MCC	FRMD4B	CPN2	AR
TBX15	SLC38A2	PDE7B	MBNL2	FREM2	CPEB3	APOF
TAPT1	SLC35D1	PDE2A	MBL2	FOXO1	COLEC11	ALDOB
SYT9	SLC31A1	PDE11A	MASP1	FOS	COBLL1	ALDH6A1
SYNPO2	SIGLEC1	PANK1	LPIN2	FAM46A	CHRM2	ALDH1B1
SULT2A1	SERPINE1	PALM2	KMO	ESR1	CDH19	ACVR1C
ST6GAL2	SERPINB9	PAIP2B	KLHL15	EPM2A	CD302	
ST3GAL6	SERPINB8	OAS2	KLF11	EPB41L4B	CD300E	
SRD5A1	RSAD2	NTN4	INMT	ELMSAN1	CCL23	
SPTBN2	RET	NRXN1	INHBC	EDNRB	CBFA2T3	
SPRYD4	RBMS3	NRBF2	IL6ST	DPYD	BMPER	

**Table 5:** GO annotation and KEGG pathway enrichment analysis of 107 potential target genes of miR-196b-5p

ID	Term	Count	%	P Value	Genes
**Biological Process**					
GO:0044712	single-organism catabolic process	19	17.8	1.94E-06	ALDH6A1, PLCXD3, SULT2A1, ALDOB, IGF1, PDE11A, KMO, CBFA2T3, LPIN2, PPARGC1A, etc.
GO:0009605	response to external stimulus	28	26.2	5.53E-05	MBL2, SLC38A2, MASP1, IL6ST, RSAD2, FOXO1, OAS2, FAM46A, CXCL12, TRIB1, etc.
GO:0044710	single-organism metabolic process	43	40.2	6.37E-05	PLCXD3, IL6ST, ALDOB, PDE11A, FOXO1, KMO, OAS2, CBFA2T3, ACVR1C, EDNRB, etc.
GO:0051094	positive regulation of developmental process	19	17.8	6.86E-05	TAPT1, AR, PTPRD, RET, CPEB3, IL6ST, AXL, IGF1, ATP11C, NRXN1, etc.
GO:1901701	cellular response to oxygen-containing compound	17	15.9	9.21E-05	RET, SOCS2, CPEB3, KLF11, ESR1, AXL, FOXO1, PPARGC1A, TRIB1, EDNRB, etc.
GO:0055086	nucleobase-containing small molecule metabolic process	15	14	0.00010186	ALDH6A1, SULT2A1, ALDOB, PDE11A, IGF1, KMO, OAS2, CBFA2T3, PPARGC1A, EDNRB, etc.
GO:0051239	regulation of multicellular organismal process	32	29.9	0.00010961	TAPT1, IL6ST, CPEB3, RSAD2, FOXO1, FAM46A, CXCL12, MEGF10, FOS, EDNRB, etc.
GO:0048806	genitalia development	5	4.7	0.00012226	AR, AXL, ESR1, SRD5A1, GJB2
GO:1901700	response to oxygen-containing compound	22	20.6	0.00012321	RET, SOCS2, CPEB3, KLF11, ESR1, AXL, FOXO1, PPARGC1A, CXCL12, TRIB1, etc.
GO:0048585	negative regulation of response to stimulus	21	19.6	0.00014142	CR1, AR, MASP1, SOCS2, IL6ST, EPM2A, ESR1, FOXO1, IGF1, NRXN1, etc.
**Cellular Component**					
GO:0043025	neuronal cell body	10	9.3	0.0006596	RET, SLC38A2, CHRM2, IL6ST, SPTBN2, PDE11A, SRD5A1, NRXN1, SLC31A1, PPARGC1A
GO:0071944	cell periphery	46	43	0.00115965	SLC38A4, SLC38A2, IL6ST, CPEB3, SYT9, MEGF10, CXCL12, ACVR1C, EDNRB, PLIN2, etc.
GO:0005615	extracellular space	19	17.8	0.00128717	MBL2, MASP1, IL6ST, AXL, IGF1, CXCL12, CPN2, SERPINB9, CCL23, BMPER, etc.
GO:0044297	cell body	10	9.3	0.00170277	RET, SLC38A2, CHRM2, IL6ST, SPTBN2, PDE11A, SRD5A1, NRXN1, SLC31A1, PPARGC1A
GO:0036477	somatodendritic compartment	11	10.3	0.00454342	RET, SLC38A2, CHRM2, CPEB3, IL6ST, SPTBN2, PDE11A, SRD5A1, NRXN1, SLC31A1, PPARGC1A
GO:0005886	plasma membrane	43	40.2	0.004909	SLC38A4, SLC38A2, IL6ST, CPEB3, SYT9, MEGF10, CXCL12, ACVR1C, EDNRB, PLIN2, etc.
GO:0097458	neuron part	16	15	0.00856024	RET, SLC38A2, CPEB3, DLGAP2, IL6ST, SYT9, PDE11A, NRXN1, PPARGC1A, FOS, etc.
GO:0044459	plasma membrane part	25	23.4	0.01229329	SLC38A4, SLC38A2, CPEB3, IL6ST, CXCL12, MEGF10, ACVR1C, EDNRB, DMD, SLC31A1, etc.
GO:0044425	membrane part	52	48.6	0.02036989	IL6ST, CPEB3, SYT9, CXCL12, MEGF10, ACVR1C, EDNRB, TMEM56, AR, RET, etc.
GO:0097060	synaptic membrane	6	5.6	0.02099924	PDE2A, CHRM2, DLGAP2, CPEB3, DMD, NRXN1
**Molecular Function**					
GO:0019199	transmembrane receptor protein kinase activity	5	4.7	0.00122381	RET, PDGFRA, AXL, TGFBR3, ACVR1C
GO:0004115	3',5'-cyclic-AMP phosphodiesterase activity	3	2.8	0.00320233	PDE7B, PDE2A, PDE11A
GO:0019838	growth factor binding	5	4.7	0.00612277	IL6ST, PDGFRA, TGFBR3, IGFBP3, ACVR1C
GO:0005102	receptor binding	17	15.9	0.00806588	MBL2, AR, PTPRD, SOCS2, IL6ST, IGF1, NRXN1, CXCL12, PRKCB, EDNRB, etc.
GO:0004114	3',5'-cyclic-nucleotide phosphodiesterase activity	3	2.8	0.01024124	PDE7B, PDE2A, PDE11A
GO:0004112	cyclic-nucleotide phosphodiesterase activity	3	2.8	0.01098852	PDE7B, PDE2A, PDE11A
GO:0008081	phosphoric diester hydrolase activity	4	3.7	0.01599651	PLCXD3, PDE7B, PDE2A, PDE11A
GO:0004118	cGMP-stimulated cyclic-nucleotide phosphodiesterase activity	2	1.9	0.01696476	PDE2A, PDE11A
GO:0030246	carbohydrate binding	6	5.6	0.01739252	MBL2, SIGLEC1, EPM2A, ALDOB, COLEC11, CD302
GO:0005126	cytokine receptor binding	6	5.6	0.01979523	CCL23, SOCS2, IL6ST, INHBC, TGFBR3, CXCL12
**KEGG pathway**					
hsa00380	tryptophan metabolism	4	3.7	0.00308774	ALDH1B1, HAAO, KMO, INMT
hsa05200	pathways in cancer	9	8.4	0.00752836	EDNRB, FOS, AR, RET, PDGFRA, FOXO1, IGF1, CXCL12, PRKCB
hsa04610	complement and coagulation cascades	4	3.7	0.01409894	MBL2, CR1, MASP1, SERPINE1
hsa00410	beta-alanine metabolism	3	2.8	0.02166778	ALDH6A1, ALDH1B1, DPYD
hsa05215	prostate cancer	4	3.7	0.02678936	AR, PDGFRA, FOXO1, IGF1
hsa05032	morphine addiction	4	3.7	0.02920649	PDE7B, PDE2A, PDE11A, PRKCB

**Note:** GO, Gene Ontology; KEGG, Kyoto Encyclopedia of Genes and Genomes.

## Abbreviations

miRNA: microRNA
HCC: hepatocellular carcinoma
qRT-PCR: quantitative reverse transcription and polymerase chain reaction
TCGA: The Cancer Genome Atlas
GEO: Gene Expression Omnibus
GO: Gene Ontology
DO: Disease Ontology
KEGG: Kyoto Encyclopedia of Genes and Genomes
PPI: Protein-Protein Interaction
SMD: Standard mean difference
95% CI: 95% confidence interval
I2: inconsistency index
AUC: area under the curve
ROC curve: receiver operating characteristic curve
SROC: summary ROC
